# Evaluating aeration and stirring effects to improve itaconic acid production from glucose using *Aspergillus terreus*

**DOI:** 10.1007/s10529-019-02742-x

**Published:** 2019-10-15

**Authors:** Nándor Nemestóthy, Péter Bakonyi, Péter Komáromy, Katalin Bélafi-Bakó

**Affiliations:** Research Institute on Bioengineering, Membrane Technology and Energetics, University of Pannonia, Egyetem ut 10, 8200 Veszprém, Hungary

**Keywords:** Aeration, Itaconic acid fermentation, Kinetic modeling, Process enhancement, Stirring

## Abstract

**Abstract:**

The effects of the bioreactor conditions, in particular the mode and intensity of aeration and mixing were studied on itaconic acid (IA) fermentation efficiency by *Aspergillus terreus* strain from glucose substrate. IA was produced in batch system by systematically varying the oxygen content of the aeration gas (from 21 to 31.5 vol% O_2_) and the stirring rate (from 150 to 600 rpm). The data were analyzed kinetically to characterize the behavior of the process, and besides, the performances were evaluated comparatively with the literature. It turned out that the operation of the bioreactor with either the higher inlet O_2_ concentration (31.5 vol% O_2_) or faster stirring (600 rpm) could enhance biological IA generation the most, resulting in yield and volumetric productivity of 0.31 g IA/g glucose and 0.32 g IA/g glucose and 3.15 g IA/L day and 4.26 g IA/L day, respectively. Overall, the significance of fermentation settings was shown in this work regarding IA production catalyzed by *A. terreus* and notable advances could be realized by adjusting the aeration and stirring towards an optimal combination.

**Graphic abstract:**

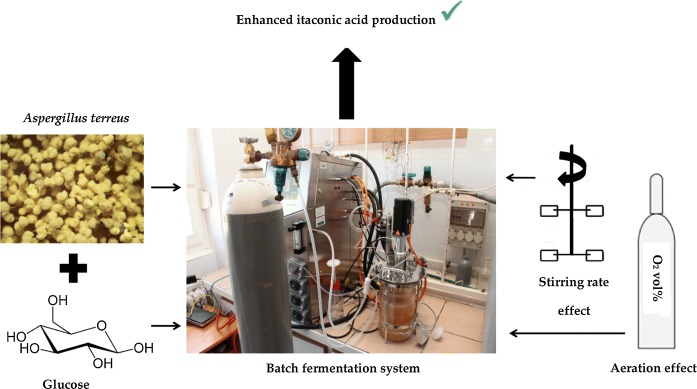

## Introduction

Whole-cell biocatalysis has been widely-employed to produce various organic components, including itaconic acid (IA) (Zhao et al. 2018). Nowadays, larger volume synthesis of IA takes place through fermentation, involving the assistance of fungal biological catalysts, in most cases strains such as *Aspergillus terreus*. In various reports, it was found that the efficiency of the process—in terms of the technologically most important IA yield and productivity—could be affected by the conditions provided in the fermenter unit. Actually, the variables that have been confirmed to take strong influence on bioreactor performance during IA production cover the quality of the medium and type of substrate, temperature, pH, aeration and stirring (Mondala 2015; Hevekerl et al. 2014; Karaffa et al. 2015; Kolláth et al. 2019; Molnár et al. 2018; Shin et al. 2013).

Recently, as part of systematic investigation, Nemestóthy et al. (2019) and Komáromy et al. (2019) showed that *A. terreus* NRRL 1960 was able to generate IA on glucose-based broth in submerged fermentation and gave positive feedback concerning the substantial role of adequate pH control strategy (Komáromy et al. 2019). By comparing the results of these studies to the literature, it could be drawn they were typically in agreement with achievements of other relevant research works (Kautola et al. 1985; Kuenz et al. 2012; Riscaldati et al. 2000). However, it should be underlined that in those previous papers from our research group (Komáromy et al. 2019; Nemestóthy et al. 2019), the experiments were carried out under fixed mixing and air supply circumstances. This means in other words that their influences on IA production capacity of *A. terreus* have not yet been addressed in our systems, although, as concluded before, proper management of these variables can enable maneuvering the process towards additional improvements (Garcia-Ochoa and Gomez 2009; Molnár et al. 2018).

Thus, in the current research, to take another step forward, IA fermentation was intended in laboratory-scale bioreactors by *A. terreus* on glucose substrate under varied operating regimes to enlighten the impact of aeration and stirring. Particularly, the effect of inlet air composition by adjusting its O_2_ content was studied in addition to that of stirring rate in the batch fermenter. The obtained data were after that analyzed and compared to the literature in order to evaluate the findings of the investigation.

## Materials and methods

### Inoculum and bioreactor set-up

The culture of filamentous fungi, *A. terreus* NRRL 1960, was used thoroughly in this work to produce itaconic acid. Strain maintenance and conditions for inoculum preparation in terms of broth composition, temperature, etc. were the same as detailed in our previous paper (Komáromy et al. 2019; Nemestóthy et al. 2019). To test the effect of varied aeration and stirring circumstances on IA production capability of the *A. terreus* strain, autoclavable Sartorius Stedim BIOSTAT® Bplus bioreactor system (with 1.5 L working volume, 10 (v/v)% inoculation ratio) was deployed (https://fenix-sd.com/content/user_files/File/Biostat_B_plus.pdf, accessed on 11. 07. 2019), employing two Rushton turbines on the central mixing shaft. The IA fermentation tests were performed under batch operating mode with the following (initial) media composition: 120 g/L glucose, 0.1 g/L KH_2_PO_4_, 3 g/L NH_4_NO_3_, 1 g/L MgSO_4_ × 7 H_2_O, 5 g/L CaCl_2_ × 2 H_2_O, 1.67 × 10^−3^ g/L FeCl_3_ × 6 H_2_O, 8 × 10^−3^ g/L ZnSO_4_ × 7 H_2_O, 15 × 10^−3^ g/L CuSO_4_ × 7 H_2_O, adopted from the recent study of Komáromy et al. (2019).

The experimental plan is shown in Table 1. As it can be seen, the impact of inlet aeration gas O_2_ content on IA production was investigated using air (21 vol% O_2_) as well as 5% and 50% O_2_-enriched mixtures (relative to the air, resulting in 22.05 and 31.5 vol.% O_2_, respectively). All gases were supplied from gas cylinders. Gas mixtures with higher O_2_ portion had been prepared using compressed (synthetic) air and oxygen (99.995% purity) (both purchased from Messer Hungarogáz Kft, Hungary) and final composition was checked and verified by GC method. Apart from that, as indicated in Table 1, the effect of stirring rate on IA fermentation efficiency was sought by varying it in the range of 150–600 rpm, employing air for this set of measurements. The pH and temperature were continuously controlled at 3 ± 0.1 and 37 °C for all the fermentations, respectively (Komáromy et al. 2019).

**Table 1:** The experimental plan performed in this study

Setting	Aeration gas flow rate (L/min)	O_2_ content of aeration gas (vol%)	Stirring rate (rpm)
1 (Control)	–	–	–
2 (Reference)	2	21	150
3	2	22.05	150
4	2	31.5	150
5	2	21	600

### Kinetic analysis of batch itaconic acid fermentations and assessment of process performance

**Fig. 1:**
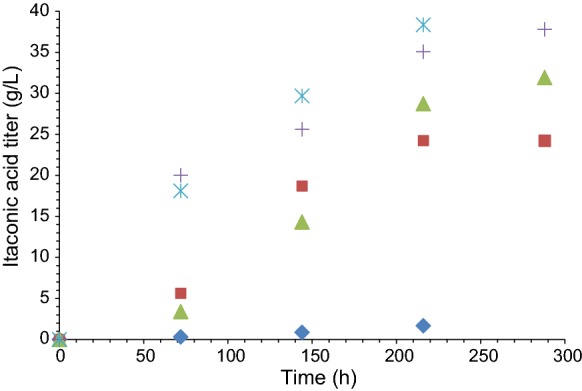
Time profiles for itaconic acid fermentation under various aeriation and mixing conditions. Symbols are according to Table 1: diamonds—setting 1 (control); squares—setting 2 (reference); triangles—setting 3; crosses—setting 4; asterisks—setting 5

The modified Gompertz-model is widely-accepted to extract useful kinetic parameters of batch fermentation and was applied with success in our previous papers on itaconic acid production (Komáromy et al. 2019; Nemestóthy et al. 2019). In general, this model (Eq. 1) with S-shape can be beneficial for the evaluation of microbiologically-catalyzed bioprocess taken into account the actual progress curves (Park et al. 2019). For the particular case of itaconic acid formation, based on the experimental data of Fig. 1, it can be applied in the following form (Eq. 1):

1 $$IA\left( t \right) \, = P\exp \{ - \exp \, [(l - t) \, + \, 1]\}$$

where IA (t), t (h), P (g/L), R_m_ (g/L–h), λ (h) and *e* represent the (i) experimental itaconic acid titer as the function of time (ii) actual process time (iii) itaconic acid titer potential (iv) maximal itaconic acid (titer) production rate (v) lag-phase time and (vi) the Euler’s number, respectively. The best model fitting could be achieved by using the Solver tool of MS Excel and the least-squares regression method. Besides, two major indices to reveal the effectiveness of IA production are the volumetric productivity (mass of IA normalized to the bioreactor working volume and the processing time in the unit of g IA/L day) and the yield (mass of IA relative to the mass of substrate ensured initially in the unit of g IA/g glucose_added_), which were therefore considered to evaluate and compare the results thoroughly similar to our previous research (Komáromy et al. 2019; Nemestóthy et al. 2019). To determine itaconic acid concentrations in samples taken at various periods of the fermentations, HPLC method as detailed by Komáromy et al. (2019) and Nemestóthy et al. (2019) was applied.

## Results

Aeration and mixing intensity are crucial factors for aerobic bioprocess optimization (Garcia-Ochoa and Gomez 2009). As the production of IA is obligatory aerobic, it requires the maintenance of sufficient oxygen supply in the whole working volume of the bioreactor, influenced by the oxygen mass transfer conditions (Shin et al. 2013). For instance, Molnár et al. (2018) proved recently that the dissolved oxygen concentration as high as 30% of saturation level notably determined the fermentation profile with *A. terreus*, particularly in terms of IA rate and yield.

In general, using mechanically-stirred bioreactors as in this study too for submerged fermentation of IA by *A. terreus*, the gas-to-liquid phase oxygen mass transfer rate is significantly dependent—besides several other parameters—on (i) how much oxygen gas is fed per unit of time and (ii) how the O_2_ is subsequently dispersed thoroughly (Garcia-Ochoa and Gomez 2009). The former can be affected by the volumetric flow rate of the inlet gas and its O_2_ content, while the latter relies strongly on the rate of stirring and were therefore examined in this work following the experimental plan in Table 1. The IA formation results are summarized in Table 2 and visualized in Fig. 1.

**Table 2:** Experimental itaconic acid concentrations obtained under the fermentation conditions listed in Table 1

Time (h)	1 (Control)	2 (Reference)	3	4	5
0	0	0	0	0	0
72	0.31	5.63	3.4	20.01	18.11
144	0.87	18.69	14.32	25.61	29.68
216	1.67	24.23	28.73	35.07	38.34
288		24.21	31.92	37.79	

As it can be noticed, the control test lacking continuous aeration and mixing produced only a negligible amount of IA, which was in agreement with the expectations and indeed pointed to the substantial role of these process variables. Compared to that, the fermentation carried out under reference settings (air, 150 rpm) was remarkably more efficient, yielding approximately 24 g/L IA concentration after 12 days. Furthermore, by assessing Fig. 1, it is clear that changing the air to O_2_-enriched aeration gas led to considerable enhancement. In this set of experiments focusing on the impact of aeration, the highest IA titer (37.8 g/L) was obtained with the gas containing 31.5 vol% O_2_. Interestingly, when the effect of stirring rate was sought in separate measurements by increasing to 600 rpm from 150 rpm, the final IA concentration was quite similar, 38.3 g/L.

**Fig. 2:**
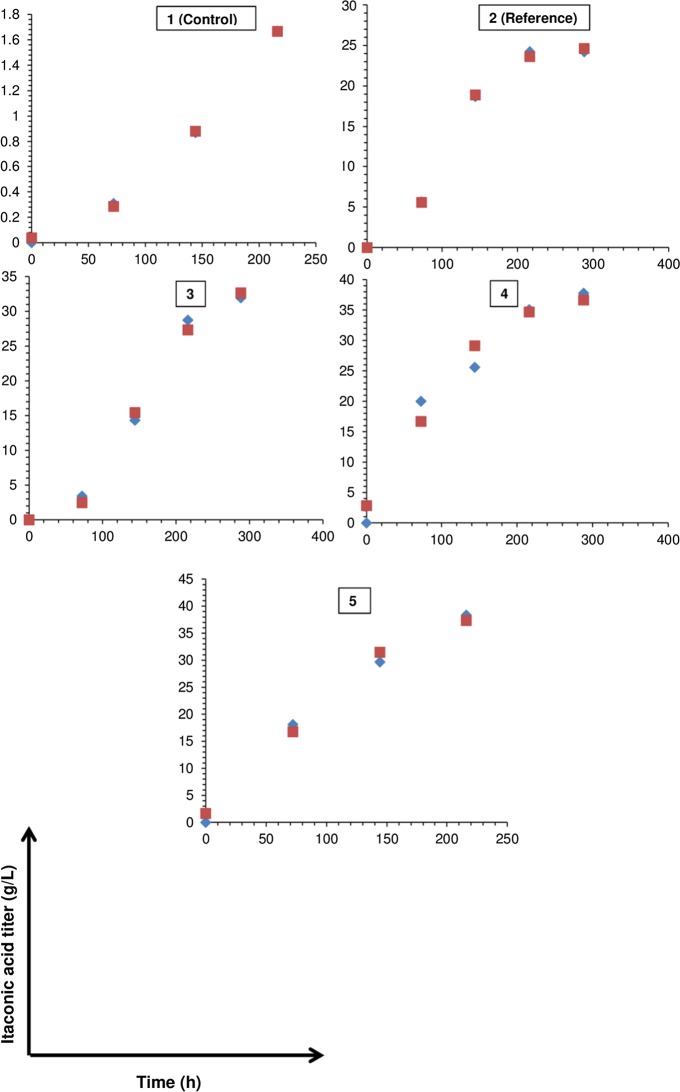
Experimental values (diamonds) versus model data (squares) using the modified Gompertz-equation for itaconic acid production

By subjecting the results in Table 2 to kinetic evaluation by the modified Gompertz-model (Sect. 2.2.), a good correlation of real and model data was found (Fig. 2) with marginal differences between maximal projected and experimental IA titers appearing in Tables 3 and 2 an, respectively, e.g. 39.8 versus 38.3 g/L (setting 5), 37.6 versus 37.8 g/L (setting 4), etc. Moreover, the explicit impact of the varied fermentation conditions on maximal itaconic acid generation rate and lag-phase time could be noted. Considering P, Rm and λ altogether to rank the fermentations (Komáromy et al. 2019), settings 4 (aeration gas with highest O_2_ content) and 5 (most intense mixing) were the most promising thanks to greater IA production capacities, rates and shortened adaptation periods.

**Table 3:** Results of the kinetic evaluation conducted on the experimental results summarized in Table 2

Model parameter	1 (Control)	2 (Reference)	3	4	5
P (g/L)	3.8	24.9	35.4	37.6	39.8
Rm (g/L–h)	0.01	0.22	0.21	0.22	0.27
λ (h)	66.1	46.7	70.8	−3.17	8.8

**Fig. 3:**
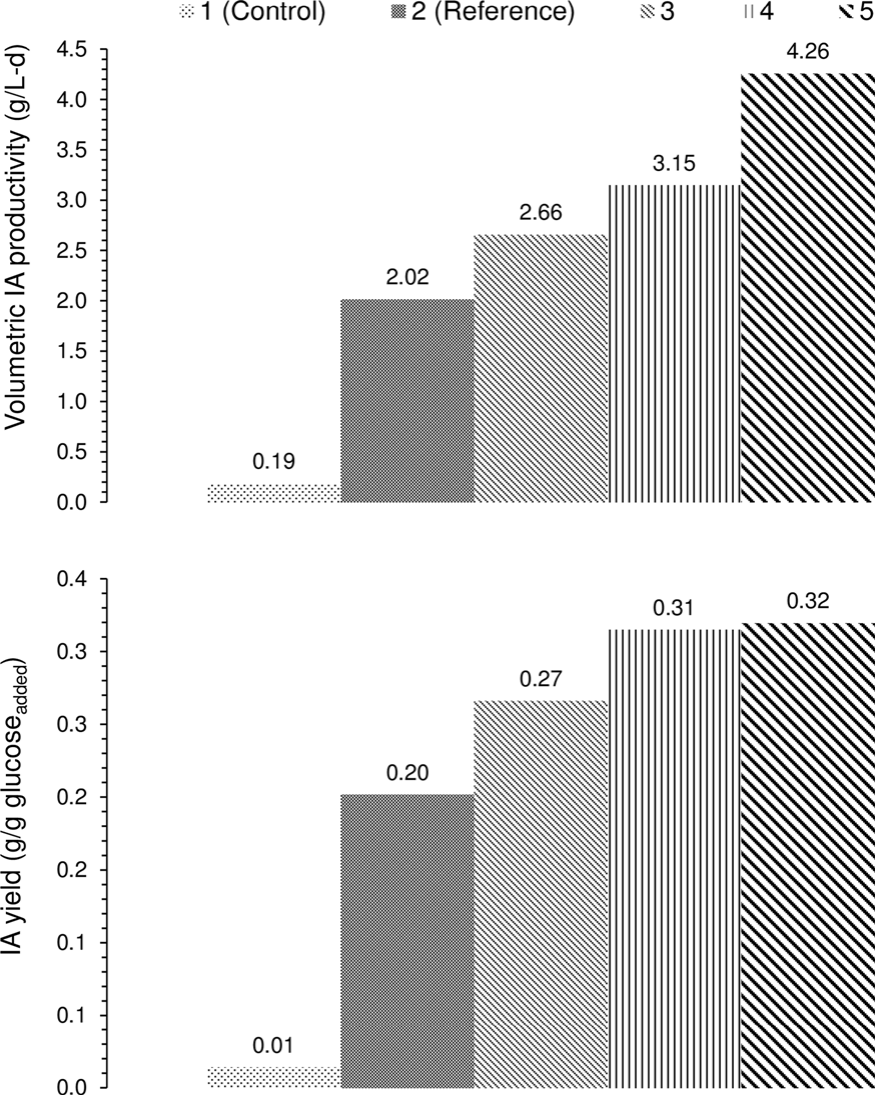
The attainable itaconic acid volumetric productivities and yields under the experimental circumstances specified in Table 1

In addition to kinetic results, the productivity and yield were computed and illustrated in Fig. 3 to assess the bioreactor performance. In our previous study 1.27 g/L day and 0.12 g IA/g glucose were achieved at pH 3, 37 °C (as in this work) at 1.5 L air/L min aeration and relatively gentle agitation (Komáromy et al. 2019). Nevertheless, in the present investigation, 4.26 g/L day and 0.32 g IA/g glucose (setting 5, Table 1) and 3.15 g/L day and 0.31 g IA/g glucose (setting 4, Table 1) could be realized. Hence, it seems that by properly improving the oxygen supplementation and mixing conditions it was truly possible to further increase the IA production performance by *A. terreus*, serving as the key-objective of this work. Further benchmarking with the already existing literature infers that the results obtained here coincide well with those presented by other scientist, covering the wider range of 2.88–12.24 g IA/L day and 0.21–0.62 g IA/g glucose (Kautola et al. 1985; Kuenz et al. 2012; Riscaldati et al. 2000).

## Discussion

**Fig. 4:**
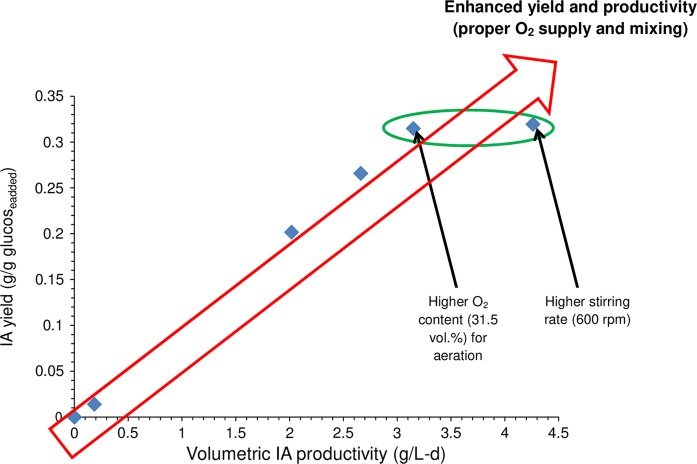
The correlation of itaconic acid volumetric productivity and yield obtained in this work

The IA yield and productivity data reported in this paper are plotted in Fig. 4 to imply that for an efficient process, it is beneficial if both of these measures are enhanced at the same time (Kumar et al. 2015).

The data within the green loop of Fig. 4—in agreement with the above discussion—illustrate that the potential of *A. terreus* to generate IA could be improved either by providing better aeration or stirring. Although in this research these two effects were distinguished, it is reasonably presumed that by combining them it will be possible to maintain better hydrodynamics and target the zone of further augmented yield and productivity (red arrow, Fig. 4). As in the aerobic bioreactor unit, the factor that takes both aeration and stirring conditions into account is the so-called K_L_a (oxygen mass transfer coefficient) (Garcia-Ochoa and Gomez 2009), the next study in our research sequence ought to deal with the examination of K_L_a in assisting IA production. For instance, Shin et al. (2013) documented the increment of K_L_a in response to more vigorous mixing (from 100 to 300 rpm) and larger air loading (0.5–1.5 L/L min) for pilot-scale IA fermenter with *A. terreus*. Nevertheless, it is to stress that simultaneous setting of aeration and mixing for favorable K_L_a mustn’t threaten the growth of *A. terreus* since the appearance of extreme shearing forces may damage its filamentous network and deteriorate biosynthesis of IA consequently.

In other words, the successful production in the bioreactor is largely dependent on higher oxygen mass transfer rate and tolerable shearing forces by microorganisms, especially in case of fungi such as *A. terreus* as pellet/filament morphology may undergo notable changes under varied stirring and aeration regimes (Casas López et al. 2005; Gao et al. 2014). Hence, morphological studies are to be performed and discussed for further elucidation of findings obtained in our fungal bioreactor.

## Conclusions

This work focused on the influence of aeration and stirring during IA production from glucose by *A. terreus* NRRL 1960. Kinetic analysis accomplished via the modified Gompertz-model verified the unambiguous effect of these two fermentation variables on IA production performance. It was found that intensified mixing and increased concentration of oxygen in the aeration gas both were in aid of realizing process enhancement. The results indicate the need for optimizing the oxygen mass transfer in the bioreactor, which can be attempted in the future (follow-up) work via the more in-depth study of K_L_a where morphological changes of fungal network are also considered for elaborating the correlations between biomass growth characteristics and metabolite production.

## Data Availability

All data generated or analyzed during this study are included in this published article.
